# Design and Development of a MOEMS Accelerometer Using SOI Technology

**DOI:** 10.3390/mi14010231

**Published:** 2023-01-16

**Authors:** José Mireles, Ángel Sauceda, Abimael Jiménez, Manuel Ramos, Rafael Gonzalez-Landaeta

**Affiliations:** 1 Applied Science and Technology Research Center, Instituto de Ingeniería y Tecnología, Universidad Autónoma de Ciudad Juárez, 450 Avenida del Charro, Ciudad Juárez 32310, Mexico; 2 Electrical and Computer Engineering Department, Instituto de Ingeniería y Tecnología, Universidad Autónoma de Ciudad Juárez, 450 Avenida del Charro, Ciudad Juárez 32310, Mexico; 3 Department of Physics and Mathematics, Instituto de Ingeniería y Tecnología, Universidad Autónoma de Ciudad Juárez, 450 Avenida del Charro, Ciudad Juárez 32310, Mexico

**Keywords:** vibration measurement, micro-opto-electro-mechanical system (MOEMS), silicon-on-insulator (SOI), Fabry–Pérot interferometry, demodulation

## Abstract

The micro-electromechanical system (MEMS) sensors are suitable devices for vibrational analysis in complex systems. The Fabry–Pérot interferometer (FPI) is used due to its high sensitivity and immunity to electromagnetic interference (EMI). Here, we present the design, fabrication, and characterization of a silicon-on-insulator (SOI) MEMS device, which is embedded in a metallic package and connected to an optical fiber. This integrated micro-opto-electro-mechanical system (MOEMS) sensor contains a mass structure and handle layers coupled with four designed springs built on the device layer. An optical reading system using an FPI is used for displacement interrogation with a demodulation technique implemented in LabVIEW^®^. The results indicate that our designed MOEMS sensor exhibits a main resonant frequency of 1274 Hz with damping ratio of 0.0173 under running conditions up to 7 g, in agreement with the analytical model. Our experimental findings show that our designed and fabricated MOEMS sensor has the potential for engineering application to monitor vibrations under high-electromagnetic environmental conditions.

## 1. Introduction

The development of monitoring technology for high-power machinery, including the conditions of harsh electromagnetic environments and machining operation applications, requires vibratory analysis as an essential tool that can be used to reduce costs and supervisory times [1,2,3,4,5,6,7]. However, most of the recent investigations on vibration analysis for applications where strong electromagnetic forces are present faced problems such as strong interface noise ratios in the sensing structures and signal-conditioning electronic interfaces [8,9,10].

Dedicated micro-electro-mechanical system (MEMS) displacement sensors are microelectronic devices that are suitable for vibrational analysis. These sensors are designed to monitor mobile MEMS components using sensing principles such as capacitance [11,12], electromagnetics [13,14], photodiode-based principles [15,16], and lasers or optics [17,18]. Each sensing principle has its own advantages and disadvantages depending on the sensing mechanism used on the micro-scale. For instance, capacitive sensors convert capacitance changes into displacement information by measuring the physical gap between the two plates. However, their performance is proportional to the plate area [11]. Electromagnetic sensors detect the intensity change of an electric current induced by a magnetic field [13]; however, a good electric shield around the engaged current-sensing element is necessary to avoid any interference in the measurement. 

In micro-opto-electro-mechanical system (MOEMS) sensors, the proof mass displacement changes the characteristics of the output light. They use several approaches, such as intensity-based detection, [3,16,19] fiber Bragg grating (FBG) [20,21], and Fabry–Pérot interferometry (FPI) [17,22,23,24,25,26,27]. An intensity-based optical displacement sensor measures the intensity change of the light and converts it into displacement information. Nevertheless, this approach is vulnerable to the stability of the laser diode and the surface conditions of the optical components. The FBG method uses a periodic modulation index inside an optical fiber or a waveguide embedded in an MEMS device. In vibration sensors using this method, either an optical fiber or a waveguide is under stress or strain owing to vibration. Thus, it changes the period of the FBG. This change can be converted into the wavelength variation of light [21]. However, the FBG requires the integration of an optical spectrum analyzer to monitor the change in the wavelength and a more complex fabrication process for the waveguide in the MEMS device. 

This research used microtechnology and an FPI. This interferometer has been widely used because of its high sensitivity, low cost, simple structure, and immunity to electromagnetic interference (EMI) [17,22,23,24,25,26,27]. We used photonics and MEMS because the merging of these technologies helps to overcome problems that are mainly found in EMI conditions, where electronic instrumentation and/or common sensors are either unable to be used or are extremely expensive.

The proposed micro-device senses the motion of a proof mass held by four designed springs. Furthermore, it uses an optical interrogation with an FPI. Other related studies have used MOEMS to measure pressure [16,20,28], strain [23,29], or in-plane motion [3,25]. However, some require a stable optical reference and no mechanical connection but still need open optical access paths to the moving target section [3,26]. To the best of our knowledge, no system has been proposed in which the optical interface is mechanically connected to a moving target and uses the structural configuration of the MEMS device. The design and development of the MOEMS sensor are described in this work. For the optical interrogation of the proof mass displacement, we developed a metal package to couple an optical fiber with the mirror section of the proof mass. In addition, we used a certified vibration system to ensure the proper characterization of the entire sensor system.

## 2. Materials and Methods

### 2.1. Sensor Structure

**Figure 1:**
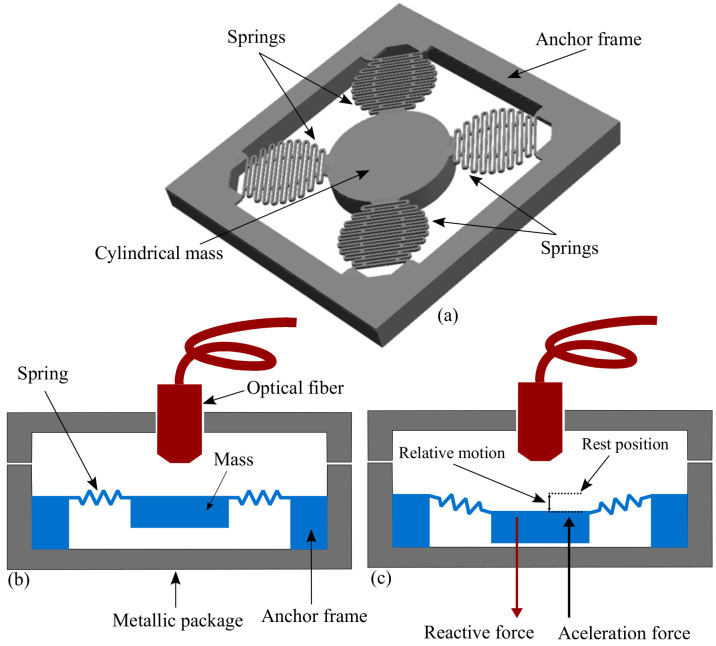
Graphical representation of (**a**) the MEMS device, (**b**) metallic package coupling the MEMS structure with an optical fiber, and (**c**) the sensor’s reaction to exerted acceleration.

**Figure 2:**
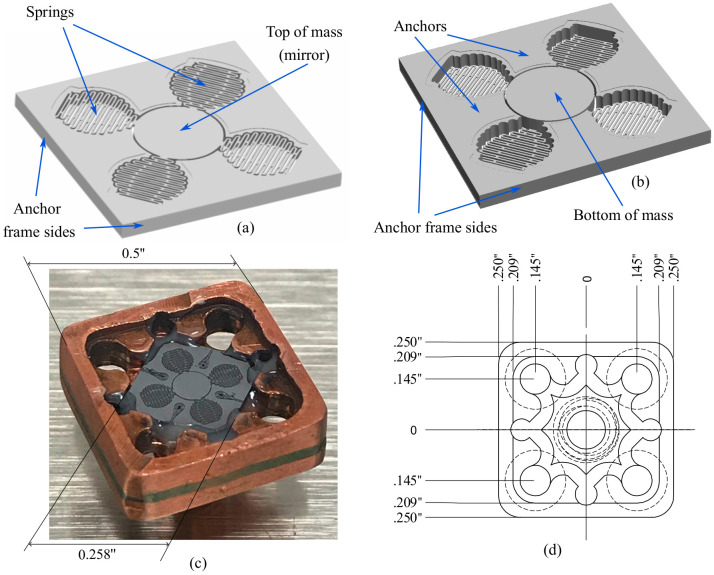
MEMS device design with the extended anchor frame showing: (**a**) the front/mirror side, (**b**) back/mounting side, (**c**) fabricated MEMS structure attached to the metallic package, and (**d**) dimensions of the metallic package.

**Figure 3:**
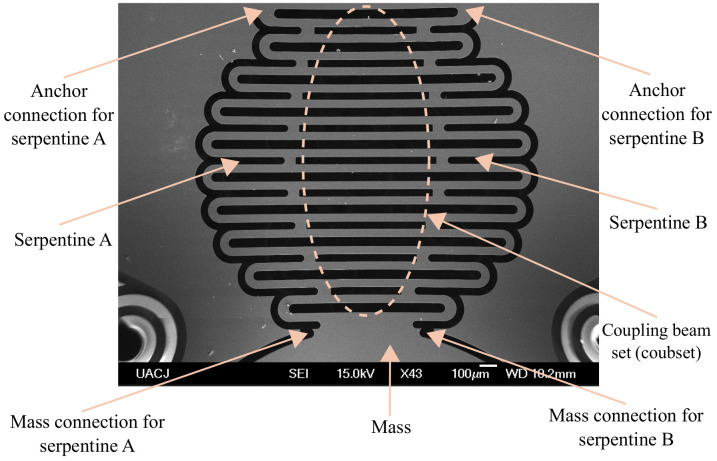
SEM image of one supporting spring of the proof mass.

The proposed sensor system consists of an MEMS device, an optical interface, and a metal package. The MEMS structure is composed of a cylindrical proof mass that is coupled with and centered among four identical springs connected to an anchor frame, as shown in Figure 1a. The anchor frame structure and optical fiber are joined to the metal package. Thus, the proof mass can be optically interrogated for displacement through an optical fiber, as shown in Figure 1b,c. The metallic package not only connects the MEMS device to an optical fiber but also protects the device. That is, it holds a ferrule from an optical fiber, in which one end tip is optically connected to the surface of the proof mass. The other end tip of the optical fiber is connected to an FPI reading system for mass-motion sensing. Figure 2 shows the front and back views of the MEMS device, with an extended anchor frame for the better support of the metallic package. The front side shows the reflective surface of the proof mass, which acts as a mirror in the interferometric reading system (see Figure 2a). The back side of the MEMS structure and the anchor sections of the extended frame are shown in Figure 2b. The bottom section of the metallic package with the MEMS sensor mounted and held with hard epoxy glue is shown in Figure 2c. Figure 2d shows the detailed dimensions of the metallic package. The top section of the package (not shown in Figure 2) holds the optical fiber end tip. Each of the four springs of the MEMS device has a set of coupled serpentine springs, as shown in Figure 3.

The novel particularity of the proposed MEMS device is its spring design and integration system. Initially, the proposed springs that we considered were serpentine-type springs (ST) with linearly scaled (LS) units, as suggested in [30]. One design considered the use of four equal ST LS springs to support a proof mass in the center. The springs were oriented to +x, −x, +y and −y with respect to the proof mass position. Nevertheless, the resulting frequencies for the non-desired modes of vibration were low and close to the desired mode (z-axis motion), owing to a simple anchor connection for each spring in either the x- or y-direction.

Therefore, the design was changed to a combination of two parallel ST springs for each of the four springs. Figure 3 shows a scanning electron microscopy (SEM) image of both the supporting spring of the proof mass and the parallel serpentine springs, denoted as A and B. Each serpentine spring is connected to one side of the proof mass, and the other side is connected to the anchor frame section. Note that the anchoring sections of springs A and B are physically more separated compared to the separation of their corresponding mass connection sections. In addition, the serpentines in Figure 3 are coupled through a beam set, named the coupled beam set or simply coubset, which connects each semicircle link of serpentine A with its corresponding semicircle link of serpentine B. This coupled, parallel configuration improves the stability of the proof mass along the z-axis.

**Figure 4:**
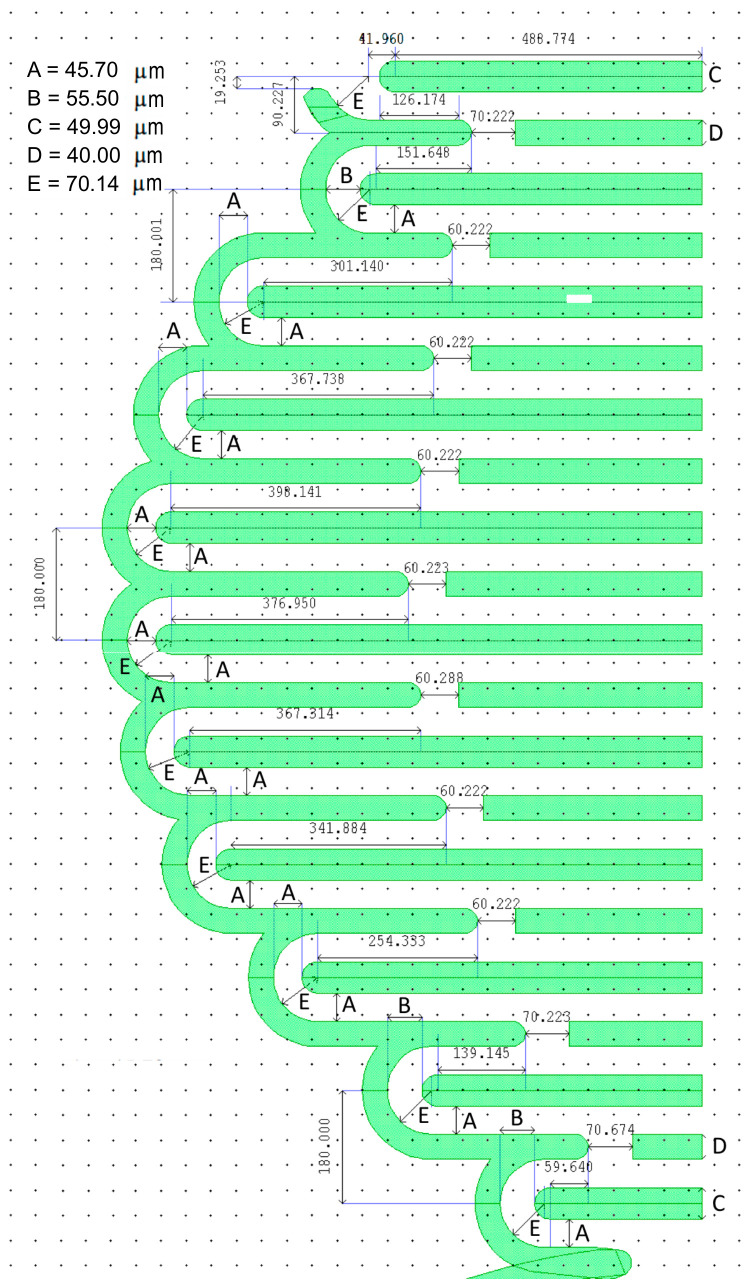
Detailed layout dimensions of the serpentine springs and half of the coubset (the color represents the etching on the device layer).

The dimensions of the serpentine sections and the coubset are shown in Figure 4. Instead of using U-springs with sharp corners in the ST springs, we used circular short links to increase the stiffness, as suggested in [30]. Most of the circular links in the serpentine use the same dimensions (denoted as A in Figure 4). However, the anchor connection section and the first two semicircular links close to the proof mass side of the serpentine were wider (length B in Figure 4). These sections represented the most highly stressed sections of the entire spring components as were detected through finite element analysis (FEA) simulations in CoventorWare^®^ 11.0, (Coventor, Raleigh, NC, USA). Therefore, they required larger dimensions.

### 2.2. Sensor Fabrication

**Figure 5:**
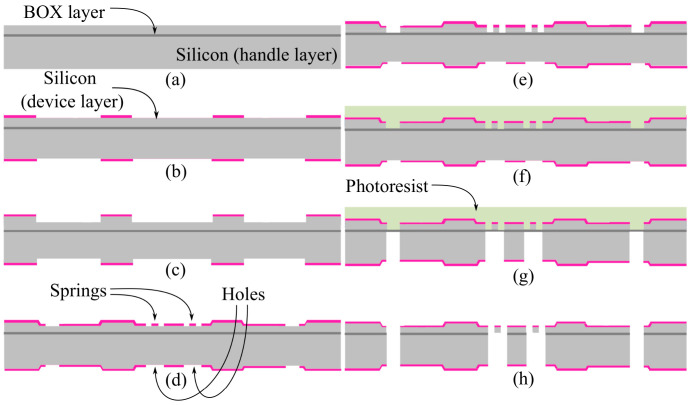
Fabrication process for the MEMS structures: (**a**) SOI wafer, (**b**) double-side alignment lithography, (**c**) device and handle DRIE process for the desired stiffness and mass weight, (**d**) lithography for the design of the springs in the device and proof mass design for the handle, (**e**) device etch for the spring machining, (**f**) device side structure protection with thick resist, (**g**) handle etch for the proof mass machining, and (**h**) BOX etch from the handle side before the resist cleaning.

The MEMS device was fabricated on a 4′′ silicon-on-insulator (SOI) wafer, as shown in Figure 5a. It had a device layer (silicon 100) of 65 μm, buried oxide (BOX) layer of 2 μm, and a handle layer (silicon 100) of 450 μm. The following steps were performed. First, the anchor structures and the desired thickness of the cylindrical proof mass were defined. This was achieved using deep reactive-ion etching (DRIE) applied to 10 μm of the silicon on the handle layer. In addition, 5 μm of silicon was machined on the device layer using DRIE, which allowed us to define the desired stiffness of the springs (this design required a 60 μm-thick spring). Double-side alignment lithography was required prior to the etching process for both the device and the handle layers. Figure 5b,c summarizes the required lithography and etching steps. The second step of the process was to clean the remains of the photoresist on both sides of the wafer and proceed with the sputter deposition of 20 and 100 nm of chrome on the device and handle layers, respectively. The chrome on the device layer served as an optical reflection layer for the mirror section of the proof mass, whereas that of the handle layer served as a hard mask for the further all-through handle DRIE machining. The third step involved the use of lithography and chrome etch processes. This step defined the structure of the sensor on the device layer, which had the shape of four springs and a central mass. Meanwhile, the central mass and holes at the bottom of the device’s layer-formed springs were defined on the handle layer, as shown in Figure 5d. The fourth step was to perform DRIE on the device layer to reach the BOX layer, as shown in Figure 5e. The thin BOX layer and springs could be broken by the helium cooling press on the back of the wafer during the etching process of the handle layer. Thus, before etching the handle layer, the fifth step was performed to protect the fragile spring structure using a thick photoresist. Figure 5f shows the 20 μm KMPR^®^ photoresist used to protect the device structure. The sixth step was to perform DRIE etching on the handle layer until it reached the BOX layer, as shown in Figure 5g. The last step had two stages. First, the BOX layer was etched using a buffered oxide etch (BOE) through the handle DRIE openings, which were defined in the sixth step. The second stage was to etch the thick photoresist using dry etching with oxygen plasma, using a flow rate of 40 standard cubic centimeters per minute (sccm) of oxygen at 25 mT, 380 W of inductively coupled plasma (ICP) power, and a 20 W radio frequency (RF) at 20 °C in a platen. 

The DRIE process conditions for the development of the MEMS device were as follows. An Oxford Instruments Plasmalab 100 system was used with 5 and 6 *s* of passivation and etching steps with 60 and 87 sccm flow rates for C(sub4)F(sub8) and SF(sub6), respectively. Both steps were performed at 28 mT, 700 W of ICP power, and 25 W of RF at 20 °C in a platen.

**Figure 6:**
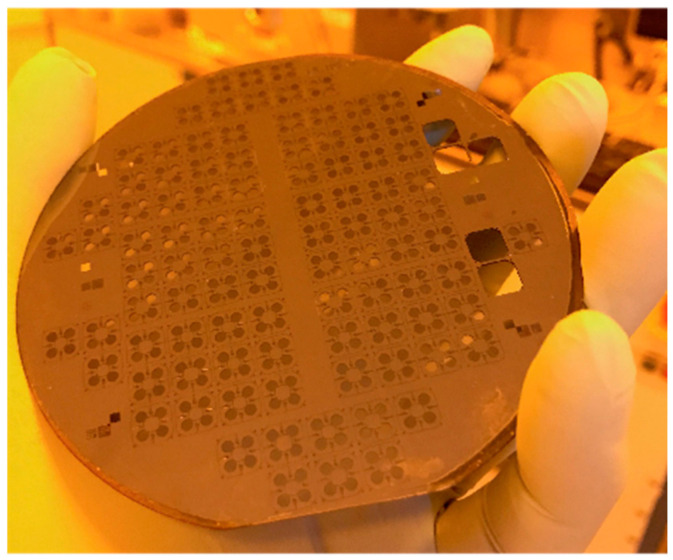
Fabricated devices on a 4″ SOI wafer.

Figure 6 shows the results of the fabrication process using a $4^{\prime \prime }$ SOI wafer. The frame of each MEMS structure was machined on the device side using the fourth step of the process, whereas on the handle side, it was machined using the sixth step. Below, all the MEMS structures are held on the wafer by easy-to-brake cantilevers, which are located at the corners of each frame. Thus, no dicing is required for the MEMS die release from the wafer.

### 2.3. Sensor Dynamic Model

**Figure 7:**
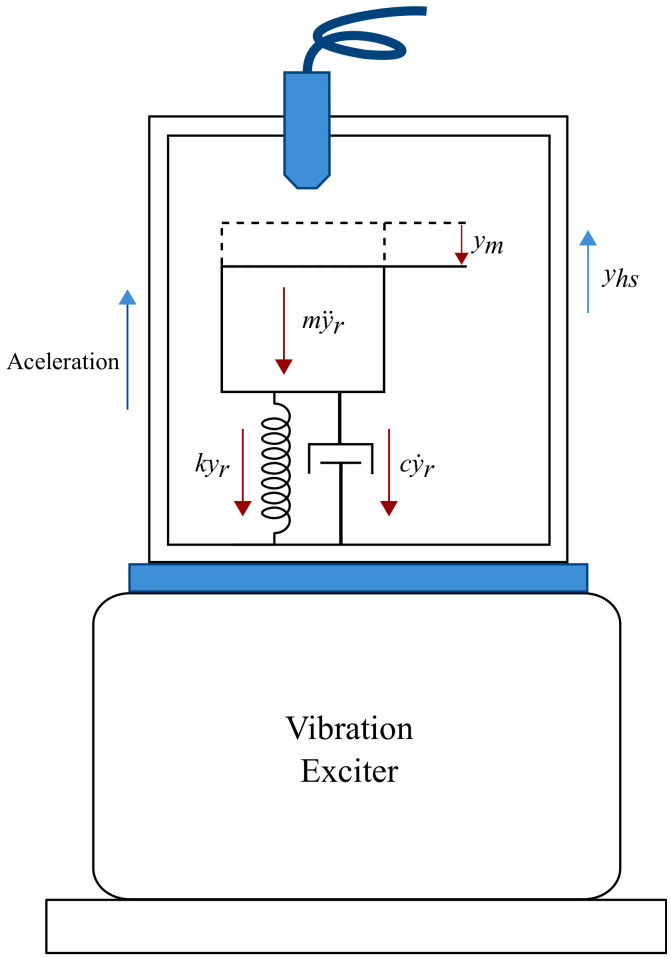
Acceleration of the metallic package and counter-reacting forces of the MEMS structures.

A dynamic model of the sensor was formulated considering the device sensor as a spring–mass–damper system. Figure 7 shows a schematic representation of a vibration shaker exerting acceleration on the metallic housing and the three resultant counterforces acting on the MEMS device. That is, when the housing is driven by acceleration in the z-direction (assuming that the mirror layer face is planar to the x−y-axes), the housing motion, denoted as $y_{hs}$, is not always the same as the proof mass motion, $y_{m}$, and the difference between the motions yields a relative motion, $y_{r}$.

Assuming that the housing motion $y_{hs}=A\sin\omega t$, $y_{r}$ is governed by Newton’s second law [31]:

(1) $$m\frac{d^{2}y_{r}}{dt^{2}}+c\frac{dy_{r}}{dt}+k_{z}y_{r}=-m\omega^{2}A\sin\omega t$$

where $y_{r}=y_{m}-y_{hs}$, m is the mass of the moving part of the device, c is the coefficient of the damper (generally, for these devices, the damping coefficient is the effect of the air or the embedded gas between the proof mass and the housing), and $k_{z}$ is the stiffness of the springs supporting the moving mass on the z-axis. A and ω are the amplitude and the angular frequency of $y_{hs}$, respectively.

We sought to restrict the performance of the sensor to frequencies lower than 800 Hz. Therefore, we set the first vibrational mode or fundamental frequency ($f_{n}=\omega_{n}/2\pi$) to 1200 Hz in the z-direction. In addition, we decided to decrease the parasitic or noisy motions of the proof mass for the associated modes of vibration on the x- and y-axes, as well as the torsional modes on the x-, y- and z-axes. Thus, we proposed the use of a spring design intended to decrease these parasitic motions (see Figure 2). They were applied to estimate the mass, spring, and damper.

We excluded the mass of the springs from the calculation of the device sensor mass. Only the proof mass was considered. We used the density of the silicon $\rho_{0}=2329\;\mathrm{kg}/m^{3}$, the radius of the cylindrical mass of 1.11 mm, and the thicknesses of the handle, BOX, and device layers of 438, 2, and 60 μm, respectively. The calculated total mass was 4.5215 mg.

**Figure 8:**
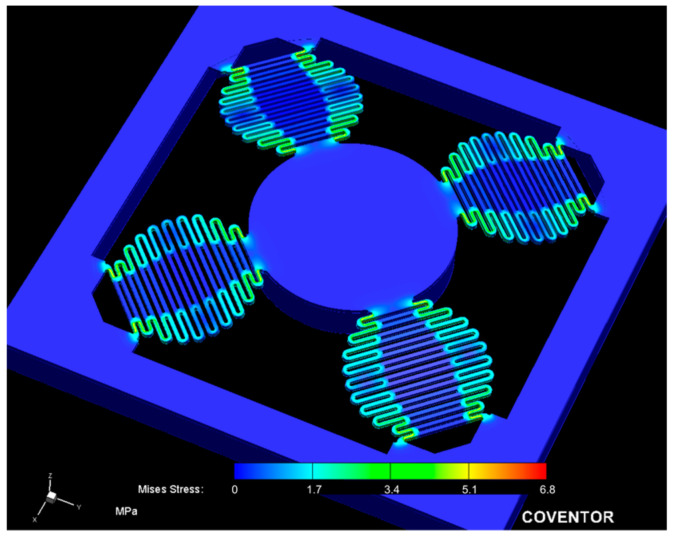
Stress-induced FEA simulation after applying force to the bottom of the proof mass.

**Figure 9:**
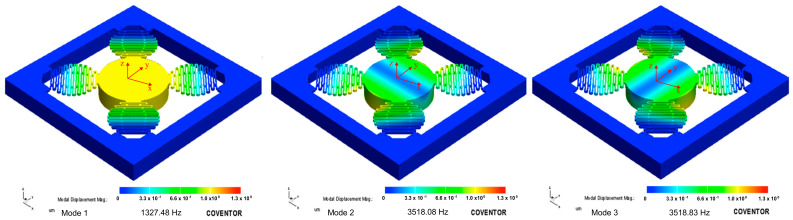
Resulting modes of vibration of the system with the coubset on the springs (shown with an exaggeration factor of 150).

The importance of the spring design is explained through a comparison of its performance with FEA simulations conducted in CoventorWare^®^ and the modal behavior of the MEMS device. The sensing device has a spring configuration that consists of four individual springs, whose combined stiffness along the z-axis is denoted as $k_{z}$. Nonetheless, as described in Section 2, each spring has a unique design consisting of two serpentine-type springs coupled in parallel through a coubset. Two spring designs, one with and one without the coubset, were simulated and compared. Figure 8 shows a stress-induced FEA simulation of the MEMS using a coubset. This result was obtained after applying a z-direction force to the bottom of the proof mass. Here, the stress is shown in a lighter color, spreading along each spring on the serpentine section. Note that there was no stress in any beam from the coubset. If the coubset in Figure 3 is not considered, the vibration of the mass–spring system exhibits three principal (lower frequencies) modes of vibration, which are important to us, since the rest appear at higher frequencies. Nevertheless, the principal modes were raised after incorporating the coubset. The frequency for the first mode of vibration with no coubset was located at 1315.89 Hz. This was very close to the first mode of vibration located at 1327.48 Hz when the coubset was used, as shown in Figure 9. Because the coubset did not significantly change the stiffness in this motion mode (as shown in Figure 8), the frequencies were similar. However, the vibration modes for the higher frequencies of 2433.08, 2433.33, and 3359.94 Hz shifted to higher frequencies when the coubset was considered. Additionally, the modes of vibration without the coubset for the frequencies 3422.60 and 3422.85 Hz were similar to those of systems with the coubset located at frequencies 3518.08 and 3518.83 Hz. As a result, the FEA simulation indicates that the coubset adds slightly more stiffness in the case of these mode behaviors, indicating a more suitable performance for the sensing strategy used in this work. 

The resulting spring stiffness $k_{z}$ was calculated using FEA by applying a total static force of 640 μN to either the top or bottom of the proof mass. The force was divided by the total mass displacement in the FEA simulations. Any force can be used if the maximum stress applied to the structures is significantly less than the yield strength of silicon. In this case, we used less than 1% of the yield strength defined by [32] or less than 50 MPa of the yield strength used by [19]. The corresponding dynamic force of 640 μN is the response for the sinusoidal input in Equation (1), using an amplitude A=1 μm and a frequency of 60 Hz in the housing. The measured thicknesses after the fabrication of the spring and proof mass were 59.75 and 438 μm for the device and handle layers, respectively. With these dimensions, using a Young modulus of 130.18 GPa ($E_{x}$, $E_{y}$, $E_{z}$) for silicon, a Poisson ratio of 0.278, and a shear modulus of 79.64 GPa, the FEA simulation result was 2.9338 μm of mass displacement. With these values, the stiffness along the z-axis $k_{z}=289.7266$ N/m was obtained.

The damping coefficient c was calculated using a combination of theoretical and experimental results. The calculus of c uses the steady-state solution for the relative motion $y_{r}$ in Equation (1), which has the following form [31]:

(2) $${\left(y_{r}\right)}_{steady}=\frac{{\left(\frac{\omega }{\omega_{n}}\right)}^{2}A\cos\left(\omega t-\phi \right)}{{\left\{{\left[1-{\left(\frac{\omega }{\omega_{n}}\right)}^{2}\right]}^{2}+{\left[2\varsigma -\left(\frac{\omega }{\omega_{n}}\right)\right]}^{2}\right\}}^{1/2}}$$

where $\omega_{n}=\sqrt{\frac{k_{z}}{m}}$, $\varsigma =\frac{c}{\sqrt{2k_{z}m}}$, ω=2πf, and ϕ denotes the phase shift of the system response. The experimental setup developed in this study used a certified accelerometer characterization system. This implies that the system uses a known acceleration in m/$s^{2}$ in the housing of the sensor. Then, using 20 m/$s^{2}$ (close to 2 g) as an input acceleration and varying the input angular frequency ω, we determined the resonant frequency ($f_{r}$). By varying ω, we also determined the maximum in the steady-state amplitude, ${\left(y_{r}\right)}_{steady}$. It is well known that for a second order system, when $\omega =\omega_{n}$ and c=0 (i.e., vacuum conditions), $f_{n}=f_{r}$, and the phase shift is 90°, which was verified by our experimental and theoretical results. In our case, the experiment was not conducted under vacuum conditions; thus, c≠0, and the phase shift at $\omega =\omega_{r}$ was lower than 90°. However, as the experiment was conducted in air, we expected the damping coefficient to be sufficiently small. Hence, we could consider $f_{r}\approx f_{n}$. Considering $\omega \approx \omega_{n}$ in Equation (2), ${\left(y_{r}\right)}_{steady}$ can be approximated as:

(3) $${\left(y_{r}\right)}_{steady}\approx \frac{A}{2\zeta }=A\frac{\sqrt{k_{z}m}}{c}$$

Experimentally, $f_{r}$ was identified at 1274 Hz, only a 5 Hz difference from the theoretical $f_{n}$, and ${\left(y_{r}\right)}_{steady}=8.97$ μm. Hence, $\omega_{r}=8004.8$ rad/s, and the controller amplitude for this frequency was calculated using an acceleration $A\omega^{2}=20$ m/$s^{2}$. Thus, from Equation (1), with $\omega =\omega_{r}$ and A=0.31213 μm, and from Equation (3), we obtained c=0.00125523.

**Figure 10:**
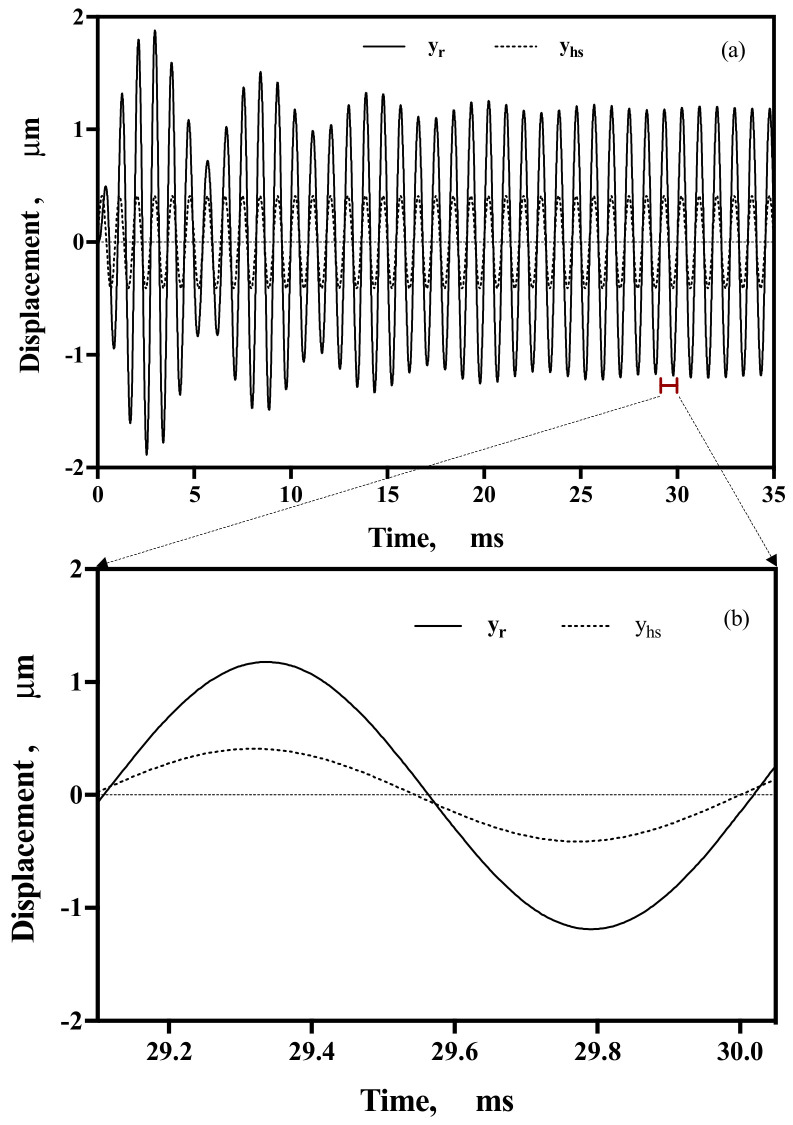
(**a**) Displacement y_rA_ (continuous line) for a reference input signal y_hs_ (dotted line) at 1100 Hz and 0.5 g of acceleration; (**b**) phase shift of 7.92 degrees detected in the last cycle from (**a**).

Having obtained all the variables from Equation (1) and using the MATLAB ode23 function, the value of $y_{r}\left(t\right)$ was obtained for any given sinusoidal input at a specific acceleration and frequency. For example, in Figure 10a, the relative mass displacement $y_{r}$ for an input signal $y_{hs}$ with a frequency of 1100 Hz and an acceleration of 0.5 g is shown. Figure 10b shows a close caption of the steady-state signal ${\left(y_{r}\right)}_{steady}$ with its phase shift. The phase shift for this model can be directly plotted using Equation (4) or estimated by comparing $y_{r}\left(t\right)$ and $y_{hs}\left(t\right)$ for a constant acceleration input, as shown in Figure 10b. The phase shift and amplitude obtained using this model were later confirmed experimentally by comparing $y_{r}\left(t\right)$ and the phase shifts.

(4) $$\phi =\tan^{-1}\left[-\frac{2\zeta \left(\frac{\omega }{\omega_{n}}\right)}{1-{\left(\frac{\omega }{\omega_{n}}\right)}^{2}}\right]$$

### 2.4. Setup Characterization

**Figure 11:**
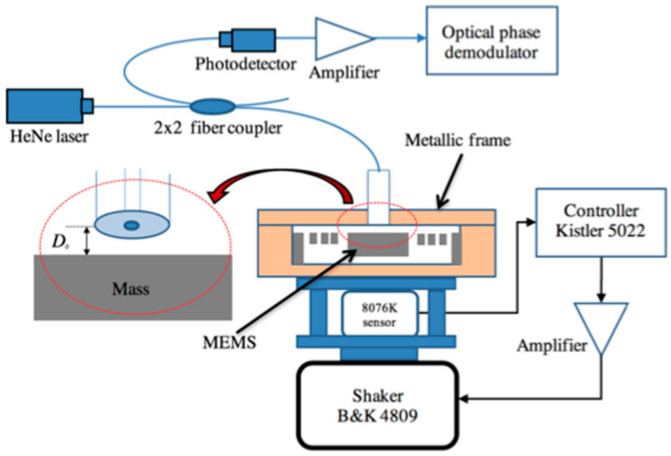
Experimental setup used to run the optical characterization of the MOEMS device.

The interrogation technique used to read the mass displacement was based on an interferometry technique, which measures the displacement $y_{r}$ using an FPI [22]. Figure 11 shows a schematic of the optical setup used for the Fabry–Pérot technique. The optical signal detected by the photodetector, assuming that all the reflection coefficients at all the optical interfaces are the same, is given by:

(5) $$I\left(t\right)=I_{0}\left[1+2\cos\left(\frac{4\pi }{\lambda }D_{x}\left(t\right)\right)\right]$$

where $I_{0}$ represents the diode laser power, λ is the diode laser wavelength, and $D_{x}\left(t\right)$ is the relative separation between the end fiber tip and the center mirror of the MEMS, which varies depending on the housing signal acceleration. In particular, if a sinusoidal signal is used to excite the MOEMS device, then $D_{x}$ is given by:

(6) $$D_{x}\left(t\right)=D_{0}+y_{rA}\sin\omega t$$

where $D_{0}$ is the initial $D_{x}$ at rest and $y_{rA}$ and ω denote the amplitude and the angular frequency, respectively. Thus, the time-varying part of Equation (5) can be expressed as:

(7) $$I\left(t\right)≅\cos\left[\frac{4\pi }{\lambda }y_{rA}\sin\omega t+\phi_{i}\right]$$

where $\phi_{i}$ is the phase difference between the interfering beams when the proof mass is at equilibrium (resting position). By expanding Equation (7) using Fourier series, considering $\left(4\pi /\lambda \right)y_{rA}\ll 1$, and setting the interferometer to $\phi_{i}=\left(\pi /2+\pi k\right)$, the amplitude and the frequency of $y_{r}$ are directly related to the electrical signal, given by:

(8) $$I\left(t\right)≅\frac{2\pi }{\lambda }y_{\mathrm{rA}}\sin\omega t$$

Nevertheless, if the vibration amplitude of the input excitation increases beyond ≅123 nm, the output sensor signal is given by Equation (7). To recover the sensor response, it is necessary to implement a demodulation method. In this work, we implemented an algorithm coded in LabVIEW^TM^, which is based on the fitting of a synthetic interferogram to the experimental one. This fitting was performed by changing the proper parameters of the amplitude and phase, considering a known input frequency. Using this demodulation scheme, we were able to demodulate input excitations with both small and large amplitudes.

**Figure 12:**
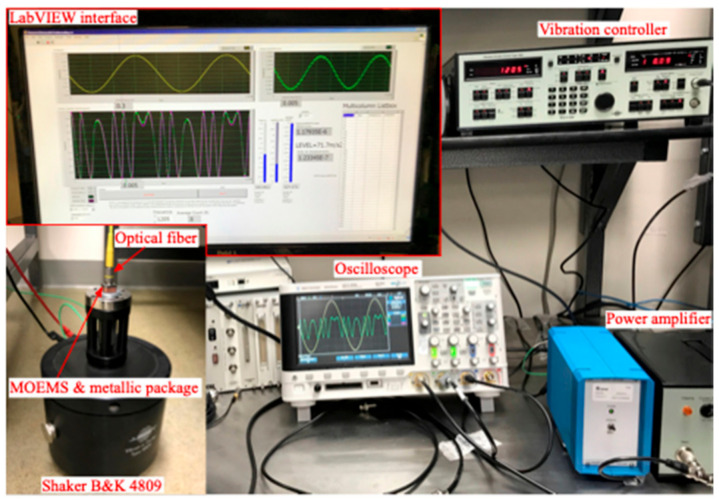
Electronic setup showing the vibration controller, oscilloscope, power amplifier, metallic package mounted on the shaker, the connector of the optical fiber (yellow), and the LabVIEW interface for the data collection and analysis.

The vibration system we used to ensure the input motion of the MOEMS sensor was a certified characterization system consisting of a Kistler 5022 controller, 8076 K reference accelerometer, and a B&K 4809 shaker. This system is supported by the Bureau International des Poids et Mesures (BIPM), the European Association of National Metrology Institutes (EURAMET), and other international associations. The system is guaranteed to range from 50 Hz to 10 kHz. The sensibility ranges from 1.022 mV/(m/$s^{2}$) at 50 Hz to 1.029 mV/(m/$s^{2}$) at 1600 Hz, showing a linear increase from 60 Hz to 1.6 kHz. Figure 12 shows pictures of the experimental setup used for the characterization of the MOEMS sensor.

**Figure 13:**
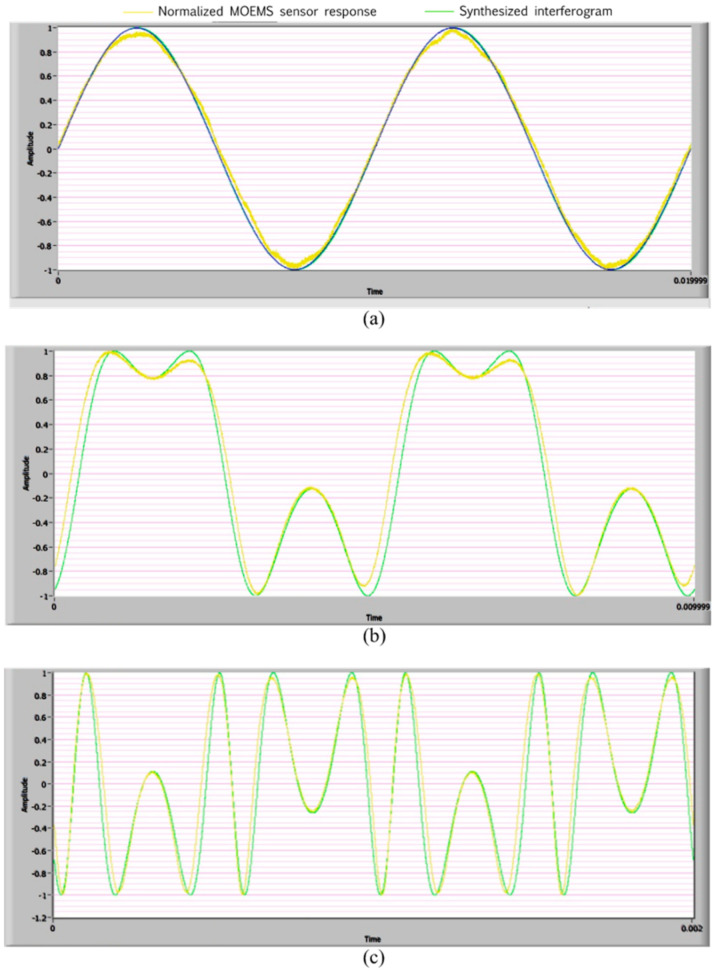
Normalized sensor response (yellow plot) and synthesized interferogram (green plot). (**a**) Sinusoidal output for an acceleration input with a sufficiently small amplitude. (**b**) Distorted sinusoidal output for an acceleration input of 20 m/s^2^ at f = 200 Hz and (**c**) f = 1000 Hz.

We systematically conducted several experiments and performed individual measurements to determine the frequency response of the sensor. In other words, we collected magnitude and phase information. First, the Bruel & Kjaer vibration control setup was used to drive the electrodynamical shaker. Then, the sensor was subjected to several acceleration inputs, and for each input, the frequency was swept. Next, each interferometric signal was captured using a data acquisition system in LabVIEW^TM^. Finally, using the experimental frequency and varying the amplitude and phase variables from Equation (3) in LabVIEW^TM^, each captured interferogram signal was matched to the greatest degree possible by plotting its corresponding synthetic interferogram, as shown in Figure 13.

## 3. Results and Discussion

The resulting die dimensions after the fabrication of the MEMS device, using an SOI wafer with 59.75 and 438 μm for the device and handle layers, were 6.391 mm × 6.391 mm. The circular mirror (top section of the mass) had a diameter of 2.22 mm. The smallest size feature of the spring structure was 45.7 μm wide.

After extensive testing, we were able to improve the performance of the first vibration mode by cancelling the vibration modes in the close range of frequencies, compared to the other designs that use standard serpentine springs. Our strategy for the demonstration of this impediment of the vibration modes was to compare the FEA simulation results of the two designs presented in Section 2. As shown in Figure 9, the modes of vibration using the coubset in the spring of this device were as follows: the first mode of vibration had a frequency of 1327.48 Hz, showing motion along the z-axis; the second mode of vibration had a frequency of 3518.08 Hz, showing slight rotational waving along the x-axis; and the third mode of vibration had a frequency of 3518.83 Hz, showing slight rotational waving along the y-axis. Thus, the use of coupled springs led to a better set of vibration modes for the application investigated in this work, whose main aim was to sense the mass motion in the z-direction (mode 1 in Figure 9).

Figure 13 shows the experimental response of the sensor and the expected interferograms. In Figure 13a, it is demonstrated that Equation (4) is valid for an input signal with a sufficiently small amplitude to obtain a sinusoidal output. This required $y_{rA}\ll 123$ nm. Figure 13b,c shows the results for the 200 and 1000 Hz excitation frequencies and the distorted sinusoidal signal, because the condition $y_{rA}\ll \frac{4\pi }{\lambda }$ was not satisfied. To convert the interferogram into an electrical signal, we used a Thorlabs photodetector model DET30B, whose responsivity at 1550 nm wavelength was 0.8 in the photoconductive mode. As can be seen in Figure 13, the photodetector signal appeared to be very similar to the synthetized interferogram. The difference between the experimental and the synthetic interferograms was due to the real sensor response, which included an additional optical phase induced by the spurious vibration of the optical fiber cable when the sensor was driven by the shaker. 

Note that we aimed to match the peaks for each shifted interferogram waveform with the experimental interferograms waveforms. To estimate the peak acceleration of the housing, we first calculated the amplitude $y_{rA}$ of the sensor and its angular frequency ω, and then we estimated the acceleration using the known terms of Equation (3) and solved the problem for A, the amplitude of the housing.

**Figure 14:**
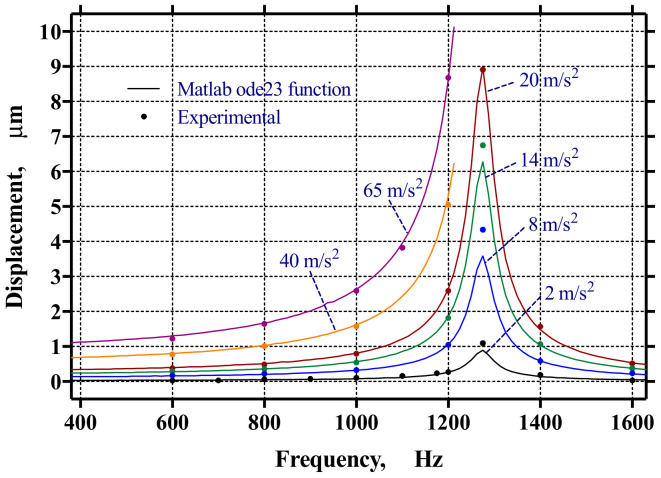
Resulting amplitude sensor response.

**Figure 15:**
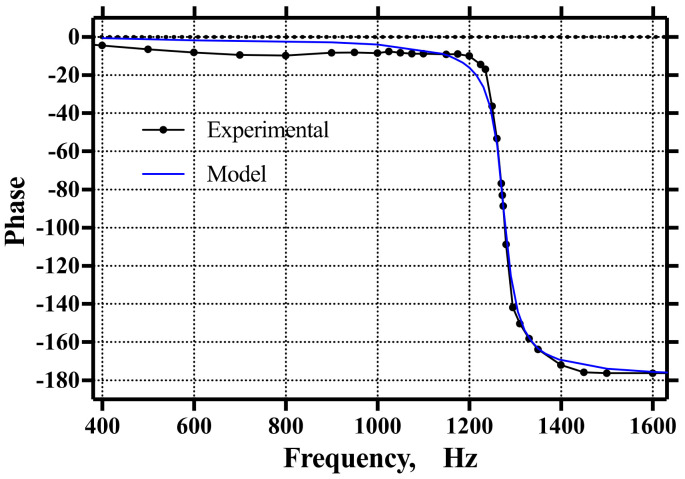
Resulting phase shift of the system with a 90-degree shift located a 1274.5 Hz.

Figure 14 and Figure 15 show the comparison between the model and the experimental results for the amplitude and phase, respectively. As can be seen, there was good agreement between the experimental data and the model. It is apparent from Figure 14 and Figure 15 that the resonant frequency $f_{r}=1274$ Hz and ${\left(y_{r}\right)}_{steady}=8.97$ μm. In addition, the system parameters identified were mass m=4.5215 mg, spring stiffness $k_{z}=289.7266$ N/m, damping coefficient c=0.00125523, and damping ratio ς=0.0173.

## 4. Conclusions

We designed, fabricated, and tested a MOEMS device consisting of a mass–spring design integrated into a metallic package and a Fabry–Pérot interferometer reading system. The fabricated MEMS dimensions were 59.75 and 438 μm for the device and handle layers, respectively. The anchor frame structure was 6.391 mm × 6.391 mm, and the circular mirror had a diameter of 2.22 mm. The MEMS device used four specially designed springs to support the proof mass. Each spring design used two novel serpentine springs in parallel and coupled through a beam set, called a coubset. The coupling of the serpentine springs showed a better performance in terms of the vibration modes for the application investigated in this work, whose main aim was to sense the motion of the proof mass in the z-direction. We used a Fabry–Pérot interferometer to experimentally read the relative displacement of the proof mass. The experimental tests were run from 0.2 g to 7 g. We obtained an MEMS model by combining experimental work, the standard spring–mass–damper second-order system model, and FEA simulations. The principal parameters of the system were obtained, including the frequency response, mass, spring stiffness, damping coefficient, and damping ratio. We compared the theoretical and experimental results, obtaining minor differences. For example, at the resonant frequency, the difference was only 5 Hz (≅0.4%). Future work will focus on developing an electronic demodulator to estimate the measurement magnitude in real time. 

## Data Availability

Data supporting this study are openly available on OneDrive at: https://alumnosuacj-my.sharepoint.com/:f:/g/personal/abimael_jimenez_uacj_mx/Ep59iTlQq0tMs6jJwVlycPoB9QazVOI6y9IAopzWWt-ZpQ?e=fZ4Epg, accessed on 27 December 2022.
